# Screening for High Intellectual Ability: Validation of the SACI Protocol and Implications for Inclusive Education in Primary Settings

**DOI:** 10.3390/jintelligence14080173

**Published:** 2026-08-01

**Authors:** Ioseba Iraurgi, Miryam Martínez-Izaguirre, Alexander Álvarez-González

**Affiliations:** 1 Faculty of Health Sciences, Department of Psychology, University of Deusto, Avda de las Universidades 24, 48008 Bilbao, Spain; ioseba.iraurgi@deusto.es; 2 Faculty of Education and Sport, University of Deusto, Avda de las Universidades 24, 48008 Bilbao, Spain; miryam.martinez@deusto.es

**Keywords:** giftedness, screening, identification, primary education, teacher nomination

## Abstract

Guaranteeing high-quality, equitable, and inclusive basic education requires upholding the right of all students to reach their full potential and ensuring their well-being. Students with High Intellectual Ability (HIA) represent a vulnerable group that is often under-identified in schools, a fact that hinders the personalization of their education and the ability to meet their specific needs. In 2019, as part of its plan to address the educational needs of this group, the Department of Education of the Basque Government prioritized the design and validation of a screening protocol to support the identification of students with HIA. This article presents a study on the validity and reliability of the High Intellectual Ability Screening Protocol (SACI, by its Spanish acronym), applicable in Primary Education. A two-phase controlled screening design was employed, involving the random assignment of participants and the use of specific statistical techniques to evaluate the psychometric soundness of the measure. The results provide highly promising evidence, supporting the conclusion that the SACI protocol is a valid and reliable screening instrument for use with Primary Education students in the Basque Autonomous Community.

## 1. Introduction

The educational interpretation of the multidimensional and dynamic nature of High Intellectual Ability (HIA), in its interaction with the environment (Dai, 2018; Sternberg, 2017), sheds light on the support provided to students who present them. It recognizes the central role of education in developing and crystallizing intellectual potential, as well as in providing care, guidance, and understanding of their needs throughout their growth process (Olszewski-Kubilius & Corwith, 2018; Subotnik et al., 2011).

The application of reliable and valid identification processes at an early age promotes respect for the educational rights of all HIA students, not only those with high performance, who are the most visible (Subotnik et al., 2011), but all who possess such abilities (Renzulli & Gaesser, 2015; Silverman, 2013). This enhances their prospects for personal, social, and academic success (Karabulut & Omeroglu, 2021) and yields clear benefits for society (EESC, 2013; Sastre-Riba & Castelló-Tarrida, 2017).

Identification has historically relied on psychometric tests that measure intelligence, achievement, aptitudes, or general ability. Although these instruments possess solid psychometric validity, they present critical limitations when transferred to diverse school contexts. On the one hand, their administration typically requires excessive time and specialized clinical training that classroom teachers lack, thereby creating a bottleneck for early detection. On the other hand, these tools must confront highly diverse sociocultural characteristics and situations of twice-exceptionality which, without a prior and deep understanding of the students’ reality, can reduce their validity when applied in a standardized manner.

Recent research confirms the suitability of teacher-based screening instruments (Biber et al., 2021; Milic & Simeunovic, 2020; Tirri, 2017) focused on learning, creativity, and the personal, social, and emotional characteristics of HIA students across diverse contexts (Elices et al., 2006). These tools should also provide significant information on skills and performance in areas often overlooked by psychometric tests, such as music, visual arts, or physical education (Wu, 2010).

There has been a notable proliferation of HIA identification protocols across different autonomous communities in Spain (Aperribai et al., 2026; Artola et al., 2003; Barraca & Artola, 2004; Hernández-Torrano et al., 2014). These consist of holistic, multi-phase models that consider personal (cognitive and non-cognitive) and contextual variables (Hernández-Torrano & Gutiérrez-Sánchez, 2014). Following a screening phase to analyze the status of the entire student body, the process moves to a specialized assessment of the intellectual profile (cognitive development, creativity, and socio-emotional aspects) (Ferrándiz et al., 2012).

The protocols implemented at a national level have attempted to address accessibility, but often at the expense of methodological robustness, relying on non-standardized qualitative observations or self-reports that lack a rigorous confirmatory factor analysis to support their structure. Furthermore, statistics provided by the Spanish Ministry of Education, Vocational Training and Sports (2024) indicate that only 0.7% of high ability students are identified, which underscores the need to continue searching for valid tools. In addition, this lack of suitable tools directly clashes with the competencies required of teaching staff. Among their essential functions is the obligation to address the diverse educational needs of all students, a responsibility that imperatively requires teachers to be capable of detecting needs linked to high abilities early on in order to articulate an appropriate pedagogical response.

To ensure effectiveness, it is necessary to establish appropriate criteria (Peters et al., 2022) and to raise awareness, train, and support teachers in their application (Laine & Tirri, 2016; Schmitt & Goebel, 2015; Vreys et al., 2018). However, obsolete and deficient teacher training in the field of high abilities, coupled with the heterogeneity of existing profiles, hinders detection and support (Roa, 2017; Schmitt & Goebel, 2015; Martínez-Izaguirre et al., 2024). Furthermore, the proliferation of official guidelines in recent years (Ferrándiz et al., 2010) has not yet reversed the low levels of identification and intervention for this group (Basque Government, 2019).

Teacher interaction with a broad range of students allows for the detection of exceptional behaviors and considers the impact of various variables (gender, language, and sociocultural factors) when analyzing strengths and both academic and non-academic achievements (Biber et al., 2021; Wu, 2010).

Teacher-led screening shows high consistency between teacher nominations and traditional achievement tests (Marsili & Pellegrini, 2022), positioning it as a valuable measure (Callahan et al., 2017; McBee, 2006). Its inclusion in identification processes allows students to be recognized as having HIA even when they might not be identified through psychometric tests alone (Jarosevich et al., 2002; Peters & Gentry, 2010; Smith, 2014). Multi-criteria identification, combining screening with formal testing, is regarded as the most appropriate practice for fostering talent identification (Marsili & Pellegrini, 2022), provided that standardized test results, which focus primarily on verbal, mathematical, or analytical domains, do not dilute the valuable information gathered through initial screening (Renzulli & Gaesser, 2015).

In its pursuit of guaranteeing educational inclusion for HIA students, the Basque Government (2019) established a strategic priority focused on the design and validation of an identification protocol to personalize educational responses. Accordingly, the objective of the present study is to validate the High Intellectual Ability Screening Protocol (SACI, by its Spanish acronym), specifically regarding its psychometric performance, construct validity, convergent validity, and discriminant validity.

However, this study is justified not only by the need to provide an operational solution to this regional administrative demand, but also by its potential to address a methodological and theoretical gap of global scope within the field of educational evaluation. The primary contribution of this study lies in the validation of the SACI protocol as a tool which seeks to harmonize practical classroom viability with maximum psychometric rigor. By overcoming the fragmentation of traditional approaches through a multidimensional model and a robust validation design, this work not only enriches research oriented toward the assessment of high ability students but also has a positive impact on school life. This instrument may support a more accessible and systematic approach to early identification by providing teaching staff with an agile and reliable resource. In doing so, it has the potential to strengthen the connection between psychometric research and educational practice, facilitating a more informed response to student diversity in real classroom settings.

## 2. Materials and Methods

### 2.1. Design and Participants

A two-phase screening design was employed for this field study (Dunn et al., 1999). In the first phase, the SACI was administered by teachers to a sample of participants within the instructional context. In the second phase, the entire group of participants from the first phase underwent a specific individual evaluation through expert assessment interviews.

#### 2.1.1. Phase One

The field study was conducted with the collaboration of the Basque Institute for Educational Evaluation and Research (ISEI-IVEI), which performed the necessary sample extraction from the census database of students in the Basque Country (BC).

The design required the differentiation of two primary groups: one composed of a random sample of the general student population in the BC (henceforth, **Random Group** or **R-G**), and another composed of a random sample of the subpopulation of students previously diagnosed with high intellectual abilities (henceforth, **HIA Group** or **HIA-G**) by a technical diagnostic team independent of the teaching staff. To control for potential selection bias identified in the literature, a third group was included, consisting of students specifically selected by their teachers (henceforth, **Nominated Group** or **N-G**).

To block the effects of sex and age, a minimum of three units of analysis was planned for each combination of conditions (Group × Sex × Age). Each comparison group consisted of 36 participants, with an equitable representation of three sampling units per grade (six grades in primary education) and sex. Consequently, the total planned sample size was 108 participants.

Based on this structure, the sample extraction was performed. For the Random Group, 36 students were randomly selected from the 2020 BAC student census (*N* = 128,152). For the HIA Group, 36 students were selected from the census of officially identified HIA students (*N* = 660), following the proposed distribution by grade and sex. For the Nominated Group, participating teachers were asked to intentionally select candidates they believed might present HIA traits based on their own criteria. In this case, the teachers were randomly selected from the teaching population to identify the participants. The application of the SACI was carried out by the teachers who were in charge of the different participating students.

#### 2.1.2. Phase Two

The objective of this phase was the assessment of intellectual abilities by an independent team of experts to establish a validated performance benchmark for comparison with the SACI results. The hypothesis was that the existing diagnosis in the HIA Group would be ratified and associated with higher SACI performance. In the Random Group, HIA identification was expected to correlate with SACI results in the same proportion, with the detection of true HIA cases being lower and close to natural prevalence. In the Nominated Group, a higher prevalence of high IQ cases was expected, associated with higher average SACI performance.

The second phase (expert evaluation) was conducted by three psychologists with Master’s degrees in General Health Psychology and sensitive to the nature of HIA students. Coordination meetings were held to unify evaluation criteria. Results were recorded in a template using the participants’ location codes.

### 2.2. Procedure

The project was collaboratively planned with officials from the Department of Education of the Basque Government (DE-GV) and the ISEI-IVEI team, who managed the official databases, sample selection, data collection, and administrative processes with participating schools.

Following the communication of the study’s objectives to school representatives, several training seminars were held for teachers on the administration of the SACI. Assessments took place the following month. Simultaneously, families were contacted to explain the study, their children’s selection, and to request informed consent. Once consent was obtained, teachers completed the assessment via a computer application. Data was managed by ISEI-IVEI and subsequently sent to the research group in an anonymized format, using identification codes managed solely by ISEI-IVEI and DE-GV technical staff.

### 2.3. Instruments

*High Intellectual Ability Screening Protocol—SACI [Screening de Altas Capacidades Intelectuales]:* The SACI is a checklist of indicators associated with HIA characteristics, developed by an expert panel based on literature evidence and existing screening programs. It is included in the Basque Government’s 2019–2022 Strategic Plan (2019). The initial 54-item checklist was refined into a 36-item short form, with seven items for each dimension: Cognitive, Learning Strategies, Creativity and Imagination, Socio-emotional, and Motivations and Interests, plus one Academic Performance indicator. Items (except academic performance) are rated on a 4-point Likert scale (Never, Sometimes, Often, Always). This checklist serves as a foundational framework for the teacher, who, after a thorough observation of the minors’ classroom activity, attitude, aptitude, and overall performance, can proceed to provide a detailed report on the frequency with which each specific item occurs. This instrument is the primary object of the psychometric and measurement performance analysis in this report.

*Battery of Differential and General Abilities—BADyG [Bateria de Aptitudes Diferenciales y Generales]* (Yuste-Hernanz et al., 2017): An aptitude test for primary and secondary students. It provides information on General Intelligence, Logical Reasoning, Verbal, Numerical, and Visuospatial factors, and IQ. It consists of 288 items across six specific subtests and three complementary ones.

### 2.4. Data Analysis

Analyses were performed using SPSS 27 (IBM Corp., 2000), FACTOR 12.04 (Lorenzo-Seva & Ferrando, 2023), and Excel for effect size calculations (Lakens, 2013).

Descriptives: Observed percentages, mean (M), standard deviation (SD), skewness (Sk), and kurtosis (K) were calculated for each item and dimension. Additionally, the Ipsative Mean Quartile (IMQ), Relative Difficulty Index (RDI), and Measure of Sampling Adequacy (MSA) were calculated to assess difficulty and convergence. The QIM index provides information regarding the quartile distribution of the mean scores; in a standard range assessment, the majority of items are expected to cluster within the central quartiles. The RDI, meanwhile, evaluates the item position; for an optimal test within a normal range, approximately 75% of the RDI values should fall within the 0.40 to 0.60 range, with the remaining values distributed uniformly across both tails of the distribution. Finally, the MSA index assesses sampling adequacy. In this regard, any value below 0.50 indicates that the item does not measure the same domain as the rest of the item set, thereby suggesting that it should be excluded from the analysis.

Reliability: The internal consistency of each dimension of the SACI scale was systematically assessed, comprising seven items per dimension, alongside the reliability of the total scale (consisting of 35 items). The contribution of each item to internal consistency was evaluated through two key indicators: the item-rest correlation (the correlation of the item with its respective scale, excluding the item itself) and the reliability of the scale, measured via Cronbach’s alpha (α), should the specific item be removed. Exclusion criteria were strictly established: an item was deemed for removal if its item-rest correlation fell below 0.40, or if the scale’s reliability improved by at least 0.05 points upon the removal of the item. Additionally, the factor loading weights of the ítems (λ) on the latent construct are presented, and the omega coefficient (ω)—a robust measure of reliability—was calculated to complement the analysis (Ventura-León & Caycho, 2017).

Dimensionality: Each construct’s dimensionality was assessed via FACTOR using polychoric correlation matrices and Item Response Theory (IRT) parameterization, specifically Samejima’s Graded Response Model (GRM). The use of the polychoric correlation matrix is highly recommended for factor analysis, particularly when dealing with ordinal items characterized by asymmetric distributions, significant excess kurtosis, or high item-rest correlation coefficients. According to Muthén and Kaplan (1992), these specific distributional anomalies can bias standard estimation procedures, making the polychoric correlation a more robust alternative for capturing the underlying relationships between items in such contexts. Parallel Analysis (PA) was used to determine the number of factors to retain (Timmerman & Lorenzo-Seva, 2011). Matrix adequacy was verified using the determinant, KMO index, and Bartlett’s test of sphericity.

Unweighted Least Squares (ULS) was the selected extraction method, chosen primarily for its robustness when dealing with polychoric correlation matrices and smaller sample sizes (Briggs & MacCallum, 2003). Furthermore, ULS is a particularly suitable approach for studies with modest sample sizes, consistently demonstrating strong performance in the context of polychoric matrices. This choice aligns with the underlying variable approach of Item Response Theory (IRT); ULS tends to yield precise estimates even in large, complex models, often outperforming more computationally intensive procedures (Lee et al., 2012). Regarding the IRT parameterization, the discrimination parameter (a) was analyzed, with values of a >0.65 serving as the threshold to indicate high discriminative capacity. In a broader sense, the item discrimination parameter reflects the item’s efficacy in differentiating between examinees with varying levels of latent ability. As this property is directly linked to the quality of the scores as a measure of the latent trait, it holds fundamental practical importance, particularly for the purposes of item selection and test refinement.

The assumption of unidimensionality was further rigorously verified using the UniCo, ECV, and MIREAL indices. These three metrics, along with their established decision criteria, served to confirm the factor structure as follows: the Unidimensional Congruence index for the scale (UniCo) and/or for the individual items (I-UniCo); the Explained Common Variance for the scale (ECV) and/or for the individual items (I-ECV); and the Mean of Item Residual Absolute Loadings (MIREAL) and/or the Item Residual Absolute Loadings (I-REAL). Following standard psychometric guidelines, values of UniCo > 0.95, ECV > 0.85, and MIREAL < 0.30 suggest that the data can be treated as essentially unidimensional, thereby supporting the use of a single-factor scoring approach.

Convergence: Convergence between SACI dimensions, total score, BADyG factors, and academic performance was assessed using Spearman’s rho (ρ) [ordinal data] or Pearsons’s r [interval data].

Discriminant Capacity: Mean differences between the three design groups were analyzed using ANOVA (Brown-Forsythe robust method if heteroscedasticity occurred). Post hoc Tukey tests were applied for significant results (*p* < .05), with Cohen’s d as the effect size estimator. Additionally, Receiver Operating Characteristic (ROC) curves were used to estimate the Area Under the Curve (AUC), Sensitivity, and Specificity, using the Youden Index (J) to determine the optimal cutoff point. Values > 80% were considered adequate (Swets, 1995).

## 3. Results

### 3.1. Descriptives

Table 1 presents the distribution data and descriptive characterization of the items and summary indices for each of the five dimensions and the total scale of the SACI. Overall, mean values are located in the center of the possible range (0 to 3), with values between 1 and 2. Only two items (Em3 and Em5) show a mean value below 1. Skewness values do not exceed unity, except for item Em5 (Sk = 1.26). In 13 of the 35 items, kurtosis values above unity with negative signs were found, indicating a platykurtic distribution. Nevertheless, RDI values for most items range between 0.40 and 0.60, and those exceeding this interval tend to balance out across both ends. Furthermore, QIM values (except for item Em5) place the means in the central quartiles, reflecting a normal range. Similarly, all MSA values are above 0.50, with the minimum value (0.73) found in item Le5.

### 3.2. Reliability

The final three columns of Table 1 provide evidence of internal consistency and reliability. No item-total correlations below 0.40 were observed, with average correlations for the different dimensions ranging from a minimum of 0.63 (Emotional scale) to a maximum of 0.77 (Cognitive scale). Moreover, the removal of any item did not improve the reliability reached by the total scales. Specifically, Cronbach’s alpha (α) and McDonald’s omega (ω) reliability values for each scale and the SACI total are very high and virtually identical across both indices.

### 3.3. Dimensionality

Table 2 presents the combined results of five Factor Analyses corresponding to each dimension-scale of the SACI protocol. In all cases, adequate factorization conditions were observed: determinants near zero (<0.0001), statistically significant Bartlett’s tests of sphericity (*p* < .001), and KMO values above 0.80. Parallel Analysis indicated the suitability of retaining a single factor in all cases; in every dimension, the eigenvalue reached by the first factor is three times higher than that of the second factor, and the explained variance by the retained factor exceeds 60%. All factor loadings (λ), except for item Le5 (λ = 0.46), show values above 0.50, ranging from a minimum of 0.55 (Item Em4) to a maximum of 0.95 (Item Mo5). Likewise, except for item Le5, the item discrimination ‘a’ values are above 0.65. Finally, regarding the UniCo, ECV, and REAL indices, some items show values outside the adequacy range (items Co6, Le5, Cr1, Cr2, Cr4, Em3, Em4, and Mo2 show at least two inadequate index values), although these indices for the total scale show correct adjustment across all scales. Taken together, these data support the hypothesis of unidimensionality for each of the SACI scales, demonstrating consistent measurement adequacy.

Going further, using the polychoric correlation matrix of the 35 SACI items, a bifactor solution with five specific factors and one general factor (GF) was programmed using FACTOR. Matrix factorization tests were adequate (determinant < 0.00001; KMO = 0.93; Bartlett’s test = 1064.9, *p* < .001), with bifactor model fit indices indicating adequacy (χ^2^ = 354.65, *p* = .949; AGFI = 0.998; CFI = 0.999; RMSEA = 0.000 [0.000 to 0.007]). Parallel Analysis suggests retaining a single factor, and the factor solution shows a higher explained variance index in the General Factor (GF = 14.12) than in the five requested factors (F1 = 2.54, F2 = 1.62, F3 = 3.76, F4 = 1.48 and F5 = 1.20). Nevertheless, factor determination indices are above 0.85 (values between 0.896 and 0.934), and the GF determination index is 0.95. ORION reliability estimates range between 0.80 and 0.87 for specific factors and 0.91 for the GF.

### 3.4. Convergence

Table 3 presents the Spearman correlation matrix between SACI sub-scales, the total scale, and related constructs. All correlations were positive and statistically significant (*p* < .001), with an average inter-scale correlation of 0.82 (range: 0.69 to 0.92) and an average scale-to-total correlation of 0.93 (range: 0.85 to 0.97). The main diagonal shows the factor loadings of the five dimensions (between 0.79 and 0.97) for a single-factor solution that accounts for 82.4% of the common variance.

The lower matrix in Table 3 shows the correlations between SACI scales and BADyG indices. Except for the socio-emotional scale, where effects were not statistically significant (*p* > .05), all other combinations showed statistical significance with moderate-to-high effects (r values between 0.40 and 0.71). The average correlation with BADyG spheres was 0.58 for the cognitive scale, 0.63 for learning strategies, 0.46 for creativity, 0.49 for motivation, and 0.57 for the total scale. Finally, correlations between SACI dimensions and academic performance also showed statistically significant results (*p* < .001) and effect sizes (values between 0.45 and 0.67, average 0.61).

### 3.5. Discriminant Capacity

The scores achieved from the SACI protocol, the BADyG, and academic performance are presented in Table 4, along with mean contrast tests and Cohen’s d effect sizes. Except for the Spatial Factor in the BADyG (F = 3.0; *p* = .064), all other contrasts were statistically significant, showing the largest effect sizes when comparing the HIA Group against the Random Group. Effect sizes for SACI comparisons between the Nominated Group and the Random Group were also notable (between 0.65 and 1.24, average 1.03), but consistently lower than those found for the HIA Group (between 1.03 and 1.49, average 1.27). SACI comparisons between the HIA and Nominated groups were not statistically significant, although moderate effect sizes were observed in two scales and the total score.

Notably, for the IQ index, all three groups differed from each other: the HIA Group had the highest mean (M = 117.5), followed by the Nominated Group (M= 106.9) and the Random Group (M = 97.3). The HIA Group showed significant and substantial effect sizes compared to the Random Group (d = 1.38) and the Nominated Group (d = 0.99). No statistically significant differences were found between the latter two, though an acceptable effect size was observed (d = 0.63). Regarding academic performance, significant differences were found for both the Nominated Group (d = 0.87) and the HIA Group (d = 0.89) compared to the Random Group, but not between the Nominated and HIA groups, which showed comparable means (Mnom = 4.17 vs. MHIA = 4.46, d = 0.06).

**Figure 1:**
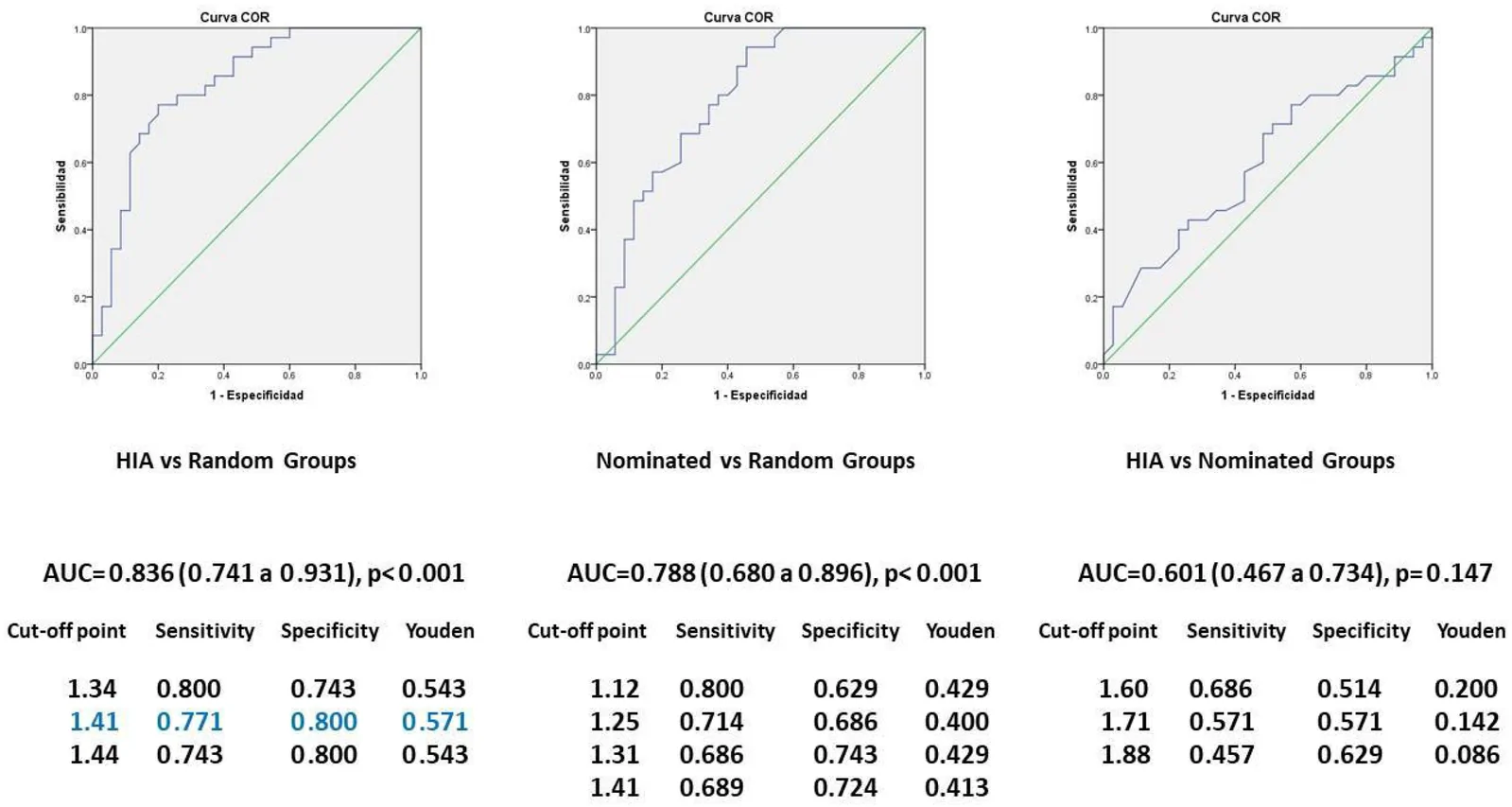
Receiver Operating Characteristic (ROC) curves for SACI protocol. [AUC (Area Under the Curve)].

Continuing the analysis of the SACI protocol’s discriminant and classification capacity, Figure 1 presents the ROC curve results. Discriminant capacity was significant for both the HIA Group (AUC = 0.836 [0.741 to 0.931], *p* < .001) and the Nominated Group (AUC = 0.788 [0.680 to 0.896], *p* < .001) relative to the Random Group, though only the former exceeded the required threshold (AUC > 0.80). Figure 1 displays sensitivity and specificity values for cutoff points that balance both criteria; however, according to the Youden index (J = 0.571), the recommended cutoff point for the total SACI index is 1.41.

## 4. Discussion

The objective of this study was to establish validity evidence for the SACI protocol, developed by the Department of Education of the Basque Government for teacher administration, to facilitate the early identification of students with High Intellectual Ability (HIA) in basic education. The results provide evidence of construct validity, discriminant capacity, convergence with related constructs, and measurement precision for both the specific subscales and the overall instrument index. While the results are encouraging, they must be considered with due caution as this is a first approach to the psychometric characteristics of the instrument. Although the sample size is acceptable (*n* = 108), representing a significant recruitment effort for this type of study, it must be acknowledged that certain limitations exist in some of the statistical procedures used, particularly regarding the factor analyses. Further studies with a larger number of participants are necessary to ratify the structural proposals introduced in this paper and to present new evidence to refine or consolidate the SACI.

The SACI was developed by a group of experts outside the research team, and the purpose was to integrate those differential, yet related, elements that are expressed in individuals with high intellectual capacities. Five theoretical areas or factors were identified (cognitive, learning, creativity, motivation, and the socio-emotional area), under the assumption that all of them could be a manifestation of a global construct, the expression of high intellectual capacity. Therefore, the purpose has been to show evidence of the psychometric behavior of each of these areas, and secondarily to test whether a common binding construct is plausible. In this way, regarding construct validity, each of the five subscales comprising the SACI demonstrated substantial unidimensionality. The items developed for each of these areas, distinct in their content, reflect and are relevant to each of the areas they assess. Reliability coefficients were optimal in all cases, with internal consistency (Cronbach’s alpha) exceeding 0.85 and omega reliability values near or above 0.90. For the total index, both values reached 0.98, indicating highly significant measurement precision. While some items exhibited atypical behavior—such as item 5 of the emotional dimension (“can be defiant with teachers”), which showed a notable floor effect—its specific content and contribution to the dimension (factor loading λ = 0.61; if item deleted α = 0.82) led to the decision to retain it. Notably, the average scores for the emotional (M = 1.18) and creativity (M = 1.36) dimensions were the lowest, both falling below the scale’s total mean (M = 1.45). These results suggest that teachers tend to award higher scores to dimensions prototypically associated with high performance (motivational, cognitive, and learning dimensions) rather than to creativity or emotional manifestations associated with HIA.

In this regard, the literature has identified potential teacher biases in screening protocols, such as the tendency to prioritize high-achieving students (Milic & Simeunovic, 2020) or those with high cognitive aptitudes, favoring rote and reproductive memory. Conversely, traits such as creativity, resistance to authority, or boredom with routine tasks are often undervalued (Barraca & Artola, 2004). This finding redefines the concept of detection bias. Rather than stemming from teacher error or a lack of attention, this detection bias reflects a limitation inherent in the educational system itself. Schools are traditionally accustomed to identifying and valuing conventional academic performance; consequently, alternative manifestations of talent tend to go unnoticed. This places the validity of the SACI protocol in a critical position, as the instrument demonstrates its capacity to capture the dissonance between teachers’ expectations regarding high intellectual ability and the actual manifestations exhibited by students.

Deficiencies in training or prejudices toward HIA students also condition identification processes (Biber et al., 2021; Barrenetxea-Mínguez & Martínez-Izaguirre, 2020). This underscores the need for prior training and awareness regarding HIA and the observation instrument itself (Karabulut & Omeroglu, 2021; Marsili & Pellegrini, 2022; Sahin & Çetinkaya, 2015), particularly concerning socio-emotional characteristics (Biber et al., 2021). Indeed, when teachers are properly trained, the validity and precision of their detection significantly increase (Barraca & Artola, 2004; Hernández-Torrano & Gutiérrez-Sánchez, 2014), positioning them as the most effective agents for identification (Biber et al., 2021). An important insight emerges from the present study. Teachers should not be viewed as static predictors but rather as dynamic variables whose judgments evolve as they develop knowledge and understanding in the field of gifted education. This finding challenges traditional models that tend to distrust teacher subjectivity and, in practical terms, indicates that screenings should not be implemented in isolation. Rather, its operational validity depends on being embedded within a process that includes prior awareness-raising and rigorous training of the professionals involved.

Concerning convergent validity, the results demonstrate a clear relationship between the SACI and other related constructs, confirming that the tool effectively addresses the target construct. A distinct feature is observed in the relationship between the socio-emotional area of the SACI and the BADyG, where no correlations of sufficient magnitude or statistical significance were found. Indeed, the socio-emotional area belongs to a different domain of assessment than that of cognitive abilities, which explains why they may exhibit orthogonality. Furthermore, the BADyG does not include any dimension that evaluates this type of expression. Nevertheless, it is an area of interest in this population, as it can intervene in the expression and development of the abilities most commonly associated with high ability (Subotnik et al., 2011), which underscores the SACI’s purpose in incorporating this type of measure.

However, the most significant evidence of the SACI’s utility lies in its discriminant capacity. Both SACI and BADyG results showed statistically significant differences for the Nominated and HIA groups relative to the Random Group, with the HIA group showing much more pronounced effect sizes (all d > 1). While teacher capacity to detect potential HIA is evident, the SACI enhances this detection (dmean = 0.50 higher in the HIA Group). Discriminant capacity assessed via ROC curves shows that the SACI discriminates better (above 80%) when comparing the Random Group to the HIA standard (AUC = 0.84) than when compared to the Nominated Group (AUC = 0.79). The optimal cutoff point established via the Youden index was 1.41, yielding a sensitivity of 0.77 and a specificity of 0.80. In contrast, using the same cutoff for the Nominated Group resulted in lower sensitivity and specificity (0.69 and 0.72, respectively), falling below the 80% standard.

A further consideration concerns the use of teacher rating scales and observation checklists in psychoeducational assessment. Although these tools are not novel in themselves, the approaches implemented to date have been characterized by two major structural limitations. First, most of these instruments lack rigorous psychometric studies supporting their construct validity, factorial structure, and discriminative capacity. Second, they have traditionally been administered in isolation, without prior teacher awareness-raising or training on how to identify the heterogeneous manifestations of HIA. This lack of teacher “visual literacy” in recognizing giftedness-related characteristics affects the quality of their observations and substantially limits both the identification rate and accuracy of screening procedures, thereby perpetuating a bias toward students with high academic achievement.

The SACI protocol addresses this scientific and operational gap through a comprehensive approach. It not only provides a tool supported by robust evidence of validity and reliability, but also conceptualizes structured observation as an inseparable component of a broader process of teacher training and awareness. By equipping teachers to recognize and interpret less conventional behaviors, such as divergent creativity or complex socioemotional profiles, the SACI protocol enhances identification at its source and prevents screening from becoming an inefficient bottleneck in the detection process.

In professional practice, the absence of a systematic screening process as a preliminary step to a more comprehensive and specific assessment has generated significant controversy. It is frequently observed that numerous cases of high ability students fail to proceed to an in-depth evaluation phase simply because they were not identified during the initial screening. This systemic failure effectively prevents these individuals from being formally recognized and, consequently, from receiving the specialized educational support they require. Therefore, it is essential to use tools with high sensitivity and psychometric rigor that do not prematurely close doors to necessary educational responses. Furthermore, if reliable tools, balancing sensitivity and specificity while correlating with psychometric tests, are not employed; screening processes may become counterproductive (McBee et al., 2016). Screening should not be relegated to a mere gatekeeping step, the information gathered is vital for identifying intellectual profiles that might be overlooked by psychometric tests where linguistic or cultural factors interfere (Sternberg, 2017). A balance between objective and subjective measures, and caution in clinical decision-making, is therefore recommended (Callahan et al., 2017; Renzulli & Gaesser, 2015).

## 5. Conclusions and Practical Implications

The present study provides empirical evidence supporting the validity and reliability of the SACI screening protocol as a tool for the early identification of students with HIA in Primary Education. The psychometric findings support the unidimensional structure of its five subscales (cognitive, learning, creativity, motivation, and socioemotional) and indicate adequate levels of internal consistency (Cronbach’s alpha ranging from 0.86 to 0.93 and McDonald’s omega between 0.89 and 0.95). In addition, the ROC analyses suggest a satisfactory discriminative capacity (AUC = 0.836 for the HIA Group and AUC = 0.788 for the Nominated Group, relative to the Random Group), identifying a cut-off score that appears to balance sensitivity and specificity effectively and may offer advantages over traditional teacher nomination procedures.

From a theoretical and methodological perspective, the findings suggest that it is feasible to combine practical applicability in educational settings with psychometric rigor in screening processes. Beyond its regional context of implementation, the SACI protocol may represent a potentially transferable framework for supporting the identification of students with diverse expressions of high ability. By incorporating dimensions that extend beyond academic achievement, such as creativity and socioemotional characteristics, the protocol may help capture profiles that are less likely to be identified through conventional referral practices. In this sense, SACI contributes to the growing body of research aimed at improving the inclusiveness and accuracy of early screening procedures for high-ability students, while further research is needed to examine its applicability across different educational contexts and populations.

The development of screening protocols to detect talent in schools is a step toward educational inclusion, enabling personalized learning that addresses the specific needs of HIA students.

By achieving this objective, this study aims to contribute to the HIA identification process, which plays a principal role for designing personalized interventions that promote talent and well-being. The next step will involve verifying the instrument’s metric behavior through systematic application and generating norms to facilitate decision-making in the specific identification and confirmation of high abilities. Likewise, further studies using the SACI with larger samples are necessary in order to ratify the findings, seeking new evidence of psychometric suitability (structural invariance, temporal consistency of its reliability, inter-rater reliability, etc.), or new evidence to help profile a more refined version of the instrument.

The validation of the SACI protocol offers several key contributions to the field of educational psychology and talent development:

*Systemic and Methodological Impact*

- Standardization of Early Detection: The SACI provides a rigorous, evidence-based tool that allows for a systematic and equitable screening process across the entire student population, reducing the reliance on unsystematic teacher observations.
- Optimization of Resources: The two-phase screening design ensures that more extensive (and costly) psychometric assessments are directed toward students who show a high probability of High Intellectual Ability, thus improving the efficiency of educational guidance services.

*Educational and Pedagogical Impact*

- Mitigation of Identification Bias: By employing a multidimensional approach (Cognitive, Creative, Socio-emotional, etc.), the protocol helps teachers identify students who may not fit the traditional “high-achiever” profile, such as those with creative or unconventional intellectual manifestations.
- Empowerment through Training: The implementation of this protocol serves as a catalyst for teacher training, fostering a deeper understanding of the diversity within HIA profiles and promoting a more inclusive school environment.
- Promotion of Data-Driven Instructional Design: The detailed item-level data provided by the SACI allows teachers to move beyond the label of ‘High Ability’ and understand the specific strengths of each student. This facilitates ‘differentiated instruction’ within the classroom, where teaching strategies can be tailored to the specific cognitive and creative profiles revealed during the screening.
- Continuous Longitudinal Monitoring: Beyond initial identification, the SACI’s modular structure allows for longitudinal tracking of a student’s development. This enables educators to observe how specific dimensions (e.g., socio-emotional vs. cognitive) evolve over time, facilitating dynamic adjustments to their Individualized Education Programs (IEP).

*Social and Policy Impact*

- Evidence-Based Policy Making: For educational administrations, the SACI offers a validated framework to monitor and improve the identification rates of HIA students, supporting the right to a personalized education through measurable and reliable data.
- Reduction of “Underachievement” and School Dropout: By identifying students whose high potential may be masked by boredom, lack of challenge, or compensatory behaviors, the protocol serves as a preventive measure against academic disengagement and underachievement, which are often prevalent among unidentified HIA students.

## Figures and Tables

**Table 1:** Characterization of SACI (Screening for High Intellectual Ability) items: Descriptive Statistics and internal Consistency.

	Items Distribution and Descriptive Statistics										Reliability		
Items	0	1	2	3	M	SD	Sk	K	RDI	MSA	r*_it_*	α	λ (ω)
Co1	8.6	31.4	32.4	27.6	1.79	0.94	−0.18	−0.97	0.59	0.88	0.75	0.92	0.85
Co2	20.0	28.6	32.4	27.6	1.50	1.02	−0.04	−1.09	0.49	0.94	0.83	0.91	0.91
Co3	21.9	34.3	21.0	22.9	1.45	1.07	0.16	−1.22	0.48	0.89	0.85	0.91	0.94
Co4	22.1	36.5	25.0	16.3	1.36	1.00	0.23	−0.99	0.45	0.91	0.71	0.92	0.79
Co5	18.1	29.5	30.5	21.9	1.56	1.02	−0.06	−1.11	0.51	0.89	0.74	0.92	0.82
Co6	11.4	21.9	36.2	30.5	1.86	0.98	−0.44	−0.81	0.61	0.86	0.73	0.92	0.82
Co7	26.7	41.9	15.2	16.2	1.21	1.01	0.51	−0.78	0.40	0.89	0.79	0.92	0.88
Cognitive					1.52	0.85	−0.07	−1.01			0.77	0.93	(0.95)
Le1	11.4	21.0	32.4	35.2	1.91	1.01	−0.50	−0.86	0.63	0.87	0.84	0.88	0.94
Le2	20.0	21.9	28.6	29.5	1.68	1.10	−0.24	−1.27	0.55	0.89	0.83	0.88	0.94
Le3	9.5	31.4	35.2	23.8	1.73	0.93	−0.16	−0.87	0.57	0.96	0.69	0.90	0.79
Le4	23.8	29.5	29.5	17.1	1.40	1.03	0.08	−1.13	0.46	0.89	0.79	0.90	0.87
Le5	29.5	41.9	15.2	13.3	1.12	0.98	0.60	−0.59	0.37	0.73	0.40	0.93	0.46
Le6	19.0	18.1	36.2	26.7	1.70	1.06	−0.35	−1.08	0.56	0.92	0.81	0.88	0.88
Le7	16.2	38.1	25.7	20.0	1.50	0.99	0.13	−1.01	0.49	0.90	0.74	0.89	0.85
Learning					1.57	0.82	−0.33	−0.99			0.73	0.91	(0.94)
Cr1	15.2	38.1	32.4	14.3	1.46	0.92	0.09	−0.78	0.48	0.85	0.73	0.90	0.83
Cr2	21.9	44.8	29.5	3.8	1.15	0.80	0.16	−0.59	0.38	0.83	0.70	0.90	0.80
Cr3	21.9	39.0	30.5	8.6	1.26	0.89	0.19	−0.74	0.41	0.75	0.78	0.89	0.88
Cr4	21.9	37.1	28.6	12.4	1.31	0.95	0.20	−0.86	0.43	0.75	0.72	0.89	0.81
Cr5	13.3	31.4	35.2	20.0	1.62	0.95	−0.11	−0.90	0.53	0.75	0.77	0.89	0.86
Cr6	15.2	29.5	31.4	23.8	1.64	1.01	−0.13	−1.06	0.54	0.93	0.72	0.89	0.81
Cr7	26.7	42.9	21.9	8.6	1.12	0.90	0.46	−0.52	0.37	0.89	0.67	0.90	0.76
Creative					1.36	0.74	−0.02	−0.92			0.73	0.91	(0.93)
Em1	18.1	33.3	27.6	21.0	1.51	1.02	0.04	−1.10	0.50	0.90	0.67	0.84	0.79
Em2	24.8	37.1	28.6	9.5	1.23	0.93	0.25	−0.82	0.40	0.91	0.70	0.83	0.81
Em3	41.3	32.7	14.4	11.5	0.96	1.01	0.76	−0.54	0.31	0.80	0.60	0.85	0.68
Em4	21.0	41.0	20.0	18.1	1.35	1.00	0.32	−0.95	0.44	0.82	0.46	0.86	0.55
Em5	57.1	30.5	9.5	2.9	0.58	0.78	1.26	1.03	0.19	0.93	0.46	0.86	0.61
Em6	24.8	37.1	21.0	17.1	1.30	1.03	0.32	−1.00	0.43	0.83	0.79	0.82	0.92
Em7	18.1	46.7	20.0	15.2	1.32	0.95	0.41	−0.67	0.43	0.90	0.73	0.83	0.85
Emotional					1.18	0.71	0.43	−0.58			0.63	0.86	(0.89)
Mo1	16.2	32.4	33.3	18.1	1.53	0.97	−0.03	−0.95	0.50	0.91	0.76	0.90	0.85
Mo2	15.2	49.5	20.0	15.2	1.35	0.92	0.44	−0.58	0.44	0.79	0.68	0.91	0.78
Mo3	12.4	38.1	29.5	20.0	1.57	0.94	0.06	−0.93	0.51	0.85	0.79	0.90	0.89
Mo4	16.2	23.8	27.6	32.4	1.76	1.07	−0.30	−1.19	0.58	0.88	0.64	0.92	0.73
Mo5	9.5	29.5	35.2	25.7	1.77	0.94	−0.22	−0.88	0.58	0.86	0.86	0.89	0.95
Mo6	16.2	35.2	28.6	20.0	1.52	0.99	0.05	−1.02	0.50	0.89	0.74	0.91	0.83
Mo7	13.3	20.0	36.2	30.5	1.84	1.01	−0.46	−0.85	0.60	0.90	0.77	0.90	0.88
Motivation					1.62	0.80	−0.17	−0.91			0.75	0.92	(0.94)
Total HIA					1.45	0.73	−0.10	−1.02				0.98	(0.98)

Notes. M: Mean; SD: Standard Deviation; Sk: Skewness; K: Kurtosis; r*_it_*: item-total correlation; α: Cronbach’s alpha if item is deleted; λ: Factor loading; (ω): Omega coefficient; RDI: Relative Difficulty Index; MSA: Measure of Sampling Adequacy.

**Table 2:** Factor solution of the SACI intelligence domains (polychoric matrices).

	Cognitive Area					Learning Strategies					Creativity and Imagination					Socio-Emotional					Motivations and Interests				
KMO (95% CI)	0.894 (0.76–0.92)					0.894 (0.73–0.90)					0.817 (0.66–0.88)					0.872 (0.68–0.87)					0.872 (0.73–0.90)				
Parallel Test	1					1					1					1					1				
Eigenvalue (1 vs. 2)	5.21/0.25					5.11/0.82					5.06/0.96					4.35/0.81					5.28/0.63				
Variance %	78.05					73.02					72.34					62.24					75.51				
Items	*λ*	a	I-U	I-E	I-R	*λ*	a	I-U	I-E	I-R	*λ*	a	I-U	I-E	I-R	*λ*	a	I-U	I-E	I-R	*λ*	a	I-U	I-E	I-R
1	0.85	1.60	0.99	0.88	0.32	0.94	2.73	1.00	0.97	0.15	0.83	1.47	0.98	0.82	0.38	0.79	1.28	1.00	0.99	0.06	0.85	1.63	1.00	0.99	0.05
2	0.91	2.21	1.00	0.99	0.03	0.94	2.71	1.00	0.97	0.16	0.80	1.35	0.97	0.81	0.39	0.81	1.37	1.00	0.99	0.03	0.78	1.23	0.92	0.71	0.53
3	0.94	2.87	1.00	0.99	0.02	0.79	1.28	1.00	0.98	0.11	0.88	1.91	0.98	0.85	0.38	0.68	0.93	0.84	0.60	0.62	0.89	1.93	0.99	0.95	0.21
4	0.79	1.31	0.99	0.92	0.23	0.87	1.78	1.00	0.98	0.09	0.81	1.36	0.96	0.79	0.43	0.55	0.65	0.91	0.69	0.38	0.73	1.05	0.99	0.88	0.27
5	0.82	1.45	0.98	0.86	0.33	0.46	0.52	0.39	0.30	0.83	0.86	1.69	0.98	0.85	0.37	0.61	0.77	0.99	0.93	0.16	0.95	3.08	0.99	0.96	0.18
6	0.82	1.45	0.98	0.84	0.37	0.88	1.89	1.00	1.00	0.01	0.81	1.36	0.99	0.89	0.28	0.92	2.30	1.00	0.99	0.07	0.83	1.49	0.99	0.90	0.27
7	0.88	1.90	1.00	0.97	0.16	0.85	1.61	0.99	0.95	0.20	0.76	1.18	1.00	0.98	0.08	0.85	1.59	0.99	0.94	0.21	0.88	1.86	1.00	1.00	0.01
Total			0.99	0.92	0.21			0.91	0.86	0.22			0.98	0.85	0.33			0.96	0.87	0.22			0.98	0.91	0.22

Notes. KMO: Kaiser-Meyer-Olkin Test; λ: factor loading; a: Discrimination parameter; I-U/I-UNICO: Unidimentional Congruence Index (>0.95); I-E/I-ECV: Explained Common Variance (>0.85); I-R/I-REAL: Residual Absolute Loading (<0.30).

**Table 3:** Correlation matrix for SACI dimensions and related constructs: Spearman’s ρ (ordinal data) and Pearson’s r (interval variables).

	Cognitive	Learning	Creativity	Emotional	Motivation	Total
SACI						
Cognitive Area	0.97					
Learning Strategies	0.91	0.93				
Creativity/Imagination	0.88	0.86	0.93			
Socio-emotional	0.78	0.69	0.77	0.79		
Motivation/Interests	0.87	0.86	0.85	0.72	0.91	
Total scale—HIA	0.96	0.94	0.94	0.84	0.93	---
BADyG						
IQ—Intellectual Quo	0.62	0.66	0.50	0.28	0.55	0.64
General Intelligence	0.67	0.69	0.53	0.24	0.57	0.66
Logical Reasoning	0.54	0.65	0.46	0.14	0.49	0.57
Verbal Factor	0.58	0.58	0.58	0.07	0.50	0.58
Numerical Factor	0.60	0.76	0.38	0.13	0.43	0.52
Visuospatial Factor	0.44	0.45	0.34	0.13	0.39	0.44
Academic Achievement	0.66	0.66	0.58	0.45	0.67	0.66

Notes. Correlation coefficients lower than 0.30 did not reach statistical significance (*p* < .05); ρ: Rho coefficient.

**Table 4:** Group Mean Differences in the SACI (Screening for High Intellectual Abilities), BADyG Performance and Academic Achievement.

Groups	Random (a)		Nominated (b)		HIA (c)		Statistic Test		Post Hoc Tests (Cohen’s *d*)		
	M	SD	M	SD	M	SD	*F*	*p*	*d*_ab_	*d*_ac_	*d*_bc_
SACI											
Cognitive Area	0.89	0.77	1.72	0.69	1.97	0.67	22.14	<.001	1.13 *	1.49 *	0.37
Learning Strategies	0.95	0.80	1.81	0.56	1.96	0.68	21.91	<.001	1.24 *	1.36 *	0.24
Creativity/Imagination	0.88	0.70	1.51	0.62	1.70	0.66	14.40	<.001	0.95 *	1.20 *	0.29
Socio-Emotional	0.81	0.60	1.24	0.67	1.49	0.71	9.52	<.001	0.68 *	1.03 *	0.36
Motivation/Interests	1.08	0.75	1.82	0.66	1.96	0.72	15.49	<.001	1.04 *	1.20 *	0.20
Total Scale—HIA	0.92	0.69	1.62	0.56	1.81	0.62	19.91	<.001	1.11 *	1.36 *	0.34
BADyG											
IQ	97.33	18.29	106.90	11.52	117.46	9.82	6.74	.005	0.63 *	1.38 *	0.99 *
General Intelligence	52.17	35.96	73.10	24.37	87.23	14.27	5.50	.011	0.66 *	1.30 *	0.71 *
Logical Reasoning	52.67	34.31	66.30	23.77	82.00	18.62	3.86	.034	0.48 *	1.08 *	0.73 *
Verbal Factor	41.75	36.36	56.20	33.55	78.62	24.82	4.31	.022	0.41	1.18 *	0.76 *
Numerical Factor	51.42	33.77	80.50	19.66	88.62	13.13	8.21	.002	1.05 *	1.45 *	0.48 *
Visuospatial Factor	61.50	27.68	63.20	28.75	83.08	15.93	3.00	.064	0.06	0.95 *	0.85 *
Academic Achievement	1.74	2.79	4.17	2.79	4.46	3.10	9.27	<.001	0.87 *	0.92 *	0.10

Note. M = Mean; SD = Standard Deviation; F: ANOVA F-statistic; *p*: *p*-value; Post hoc effect size: Cohen’s *d* coefficient *: Statistically significant sizes; Cohen’s d values > 0.80 are considered large effects; values between 0.60 and 0.80 are considered moderate-to-large effects.

## Data Availability

The data that support the findings of this study are available from the corresponding author upon reasonable request.

## Abbreviations

The following abbreviations are used in this manuscript:

SACI	High Intellectual Ability Screening Protocol—Screening de Altas Capacidades Intelectuales
HIA	High Intellectual Abilities
BADyG	Batería de Aptitudes Diferenciales y Generales (Battery of Differential and General Abilities)
ISEI-IVEI	Instituto Vasco de Investigación y Evaluación Educativa (Basque Institute for Research and Evaluation in Education)
